# PHYSICAL ACTIVITY, SEDENTARY TIME, AND ASSOCIATED FACTORS IN POST-COVID-19 CONDITION: A CROSS-SECTIONAL STUDY

**DOI:** 10.2340/jrm.v57.43967

**Published:** 2025-10-22

**Authors:** Anna TÖRNBERG, Anna SVENSSON-RASKH, Lucian BEZUIDENHOUT, David Moulaee CONRADSSON, Annie SVENSSON, Judith BRUCHFELD, Elisabeth RYDWIK, Malin NYGREN-BONNIER

**Affiliations:** 1 Department of Neurobiology, Care Sciences and Society, Karolinska Institutet, Huddinge, Sweden; 2 Medical Unit Allied Health Professionals, Karolinska University Hospital, Stockholm, Sweden; 3 Department of Health and Rehabilitation Sciences, Division of Physiotherapy, Stellenbosch University, Cape Town, South Africa; 4 Department of Medicine, Solna, Karolinska Institutet, Stockholm; 5 Department of Infectious Diseases, Karolinska University Hospital, Stockholm, Sweden

**Keywords:** fitness trackers, health-related quality of life, mental health, physical activity, physical fitness, post-acute COVID-19 syndrome, postural orthostatic tachycardia syndrome, sedentary behaviour

## Abstract

**Objective:**

This study aimed to explore physical activity and sedentary time in adults with post-COVID-19 condition, and to identify associated factors.

**Design:**

Cross-sectional, observational study.

**Subjects/Patients:**

Adults with post-COVID-19 condition.

**Methods:**

Physical activity and sedentary time were measured using activity monitors alongside assessment of potential associated factors.

**Results:**

Among 159 participants (mean age: 50 years, women: 64%), 36% took < 5,000 steps, 60% spent < 22 min in brisk walking, and 57% spent ≥ 8 h sedentarily daily. Additionally, 29% exhibited low activity combined with prolonged sedentary time. Postural orthostatic tachycardia syndrome and palpitations were associated with taking fewer steps, while paraesthesia, greater distance in the 6-min walk test, previous activity levels, and self-rated health were associated with taking more steps. Palpitations were associated with less brisk walking, whereas greater distance in the 6-min walk test and lung function were associated with more brisk walking. Postural orthostatic tachycardia syndrome was associated with increased sedentary time and with exhibiting low activity combined with prolonged sedentary time.

**Conclusion:**

A substantial proportion of individuals with post-COVID-19 condition exhibit low physical activity and prolonged sedentary behaviour, posing potential health risks. The associated factors underscore the importance of comprehensive assessments to inform safe, individualized interventions.

## LAY ABSTRACT

After having COVID-19, many people continue to live with long-term symptoms, described as long COVID, that impact their everyday lives. In this study, we looked at how physically active participants with long COVID are, and how much time they spend sitting still. We used activity monitors to measure movement and sitting time. We found that many participants were not very active and spent large parts of the day being inactive. Some symptoms, such as feeling dizzy when standing up or having a racing heart, were linked to lower activity levels and more sitting time. Participants who were in better physical shape or had been more active before becoming ill tended to move more. These findings highlight the need to support people with long COVID in safely increasing their activity levels. Understanding how different symptoms affect movement can help healthcare professionals provide better, more personalized advice.

Post-COVID-19 condition (PCC) is an umbrella term that describes persistent and heterogeneous symptoms and impairments affecting 1–6% of individuals who previously had COVID-19. PCC potentially impacts hundreds of millions worldwide and hundreds of thousands in the Nordic countries, and most individuals now living with PCC were not hospitalized due to COVID-19 (1, 2). Factors such as being bedridden for a week or longer, female sex, being unvaccinated, recurrent COVID-19 infections, and pre-existing comorbidities increase the risk of developing PCC (1, 3). Though PCC severity varies, about 20% of affected individuals report severe symptoms often across several organ systems (1). Common symptoms and impairments include fatigue, dyspnoea, impaired aerobic capacity, pain, depression, and post-exertional symptom exacerbation, which refers to the worsening of symptoms 12–72 h after physical or mental exertion (1). Additionally, about 30% of individuals with severe PCC develop postural orthostatic tachycardia syndrome (POTS), a severe form of autonomic dysfunction that causes symptoms of orthostatic intolerance such as dizziness, palpitations, tremors, weakness, and blurred vision (4).

To date, research suggests that PCC may lead to limitations in physical activity and increased sedentary time (5–7). For physical activity, both number of steps and time in moderate-to-vigorous physical activity (MVPA) seem to be affected (5, 6). A few studies have shown that female sex, fatigue, and post-exertional symptom exacerbation are associated with taking fewer steps and spending less time in MVPA, while factors associated with sedentary time remain unclear (5, 7, 8). Although previous research has examined the impact on physical activity and sedentary time, no study has specifically addressed the combined impact on these behaviours. A low physical activity level and prolonged sedentary time, especially when combined, have been associated with an increased risk of numerous chronic diseases and all-cause mortality in the healthy adult population (9–11). Further research on physical activity and sedentary behaviour is necessary to better understand the diverse factors affecting individuals with PCC. Expanding this knowledge could inform recommendations and targeted interventions for physical activity, potentially leading to improved health outcomes.

The aim of this exploratory study was twofold. First, the study aimed to describe device-assessed physical activity, including the number of steps and brisk walking time, as well as sedentary time among adults with PCC. Second, the study aimed to identify factors associated with the number of steps, brisk walking time, sedentary time, and exhibiting low physical activity combined with prolonged sedentary time.

## METHODS

### Study design, setting, and participants

This prospective, observational, cross-sectional study is part of the ReCOV research project (Recovery and rehabilitation during and after COVID-19), for which a study protocol has been published elsewhere (12). ReCOV is a collaboration between several departments at the Karolinska University Hospital and Karolinska Institutet in Stockholm, Sweden. Participants in ReCOV are primarily recruited from the specialized, interprofessional post-COVID outpatient clinic at Karolinska University Hospital. At this clinic, assessments are conducted on adult individuals (≥ 18 years), either referred following hospitalization due to COVID-19 or those with severe PCC who were not hospitalized, referred from primary care or self-referred. Referral criteria for previously hospitalized individuals include treatment in the intensive care unit, requiring at least 2 L/min of oxygen, extensive pulmonary radiographic changes, and/or a complicated clinical course. For non-hospitalized individuals, criteria include the World Health Organization’s definition of PCC and severe functional impairments causing at least 50% disability or sick leave (13). All patients attending the post-COVID clinic were considered eligible and were asked to participate in the ReCOV project during their clinic visits. For the present study, patients with a visit during certain periods between December 2020 and August 2024, primarily during most of 2021 and 2022, were also asked to wear an activity monitor. Due to staff availability and the accessibility of activity monitors, data collection was not possible at all times.

### Procedure and outcome measures

Physical function, self-reported physical activity, mental health, and self-rated health were assessed by physiotherapists and psychologists at the post-COVID clinic. This was followed by device-assessed physical activity and sedentary time in the home environment. Demographic and assessment data were collected from medical records and managed using REDCap (https://project-redcap.org/) electronic data capture tools hosted at Karolinska Institutet.

*Demographic data* included information on sex, age, body mass index (BMI), education, previous and current employment rates, smoking status, previous comorbidities, hospitalization status during COVID-19, time since COVID-19, diagnosis of POTS related to COVID-19, Post-COVID-19 Functional Status Scale, and self-reported symptoms.

*Physical function* was assessed using the 6-min walk test (6MWT), the 1-min sit-to-stand test, a hand grip strength test, the maximal inspiratory pressure test to measure inspiratory muscle strength, and dynamic spirometry to measure forced expiratory volume in 1 s and forced vital capacity. All tests were performed according to guidelines and are presented as a percentage of the predicted value (14–20). Additionally, dyspnoea was assessed using the Modified Medical Research Council dyspnoea scale, ranging from 0 to 4, where a score of 2 or higher indicates clinically relevant dyspnoea (21).

*Self-reported physical activity* was assessed using the Frändin/Grimby activity scale, which ranges from 1 (“hardly any physical activity”) to 6 (“hard or very hard physical activity regularly and several times a week”) (22).

*Mental health* was assessed by measuring symptoms of depression and anxiety using the Patient Health Questionnaire-9 (PHQ-9) and the Generalized Anxiety Disorder 7-item scale (GAD-7) (23, 24). The PHQ-9 score ranges from 0 to 27, and the GAD-7 score from 0 to 21. Higher PHQ-9 or GAD-7 scores indicate more severe symptoms of depression or anxiety, and a score of 10 or above is considered to indicate moderate to severe depression or anxiety (23, 24).

*Self-rated health* was measured using the EQ VAS in the EQ-5D instrument, where individuals rate their health from 0 (“the worst health you can imagine”) to 100 (“the best health you can imagine”) (25).

*Physical activity and sedentary time* were measured using the activPAL™ activity monitor (version 3 or 4, PAL Technologies Ltd, Glasgow, Scotland). The activPAL is a triaxial accelerometer validated and reliable in healthy adults, which classifies posture (sitting/lying vs standing/stepping) and records step counts and cadence based on thigh-worn accelerometery (26). The activity monitor was placed on the participant’s anterior thigh with a waterproof covering and adhesive film. Participants were instructed to wear the monitor for at least 7 consecutive days, removing it only for baths or saunas, noting removals in a diary, and returning it by mail after measurements. Activity data were then exported as 15-s epoch CSV files using the CREA algorithm (version 1.3) in PAL software tools (https://www.palsystem.com/en/products/pal-system-software/) and imported into RStudio (R Foundation for statistical Computing, Vienna, Austria) for wear time validation and analysis. During data inspection, it became evident that the CREA algorithm did not consistently identify awake time across all participants. Therefore, to more accurately determine awake periods, specific criteria based on previously described methods were applied (27). The onset of an awake period was defined as the first 30-min activity bout (> 70 steps and/or > 10 min upright) between 04:00 and 12:00, following at least 2 h of sedentary time. The end of an awake period was defined as the first 90-min sedentary bout (< 50 steps and/or < 5 min upright) after 22:00. If no onset or end of awake periods could be identified, 10:00 and 22:00 were used as defaults. Participants with at least 10 h of awake wear-time per day for at least 5 days were included in the analysis.

Measures used to describe physical activity and sedentary time included the number of steps, brisk walking time, and awake sedentary time (i.e., sitting or lying down). Brisk walking time, used as a proxy of time in MVPA, was defined as a stepping cadence of at least 100 steps per minute or 1.67 steps per second (28). To determine brisk walking time, the stepping cadence was calculated by dividing the number of steps taken by the time spent stepping for each 15-s epoch (29). The total brisk walking time per day was then obtained by summing the stepping time in all 15-s epochs with a cadence of at least 1.67 steps per second. Participants who took fewer than 5,000 steps per day and engaged in less than 22 min of brisk walking, corresponding to less than 150 min per week, were classified as having low physical activity (10, 11). Those who spent more than 8 h per day in sedentary behaviour were classified as having prolonged sedentary time (30). Additionally, we reported the number of participants who exhibited both low physical activity and prolonged sedentary time.

### Statistical analysis

Statistical analyses were performed using RStudio. Data were described using mean (SD), median (IQR), or frequency (*n*) and valid proportion (%), as appropriate. The level of significance was set at *p* < 0.05 for all analyses. For the first aim, number of steps, brisk walking time, and sedentary time, as well as the frequency and proportion of participants with low physical activity and prolonged sedentary time, were described. For the second aim, 3 separate multiple linear regression analyses were conducted to identify associated factors, using average daily steps, brisk walking time, and sedentary time as dependent variables. Additionally, a multiple logistic regression analysis was performed to identify associated factors, using low physical activity combined with prolonged sedentary time as the dependent variable. A total of 38 possibly associated variables were selected as potentially independent variables for the regression models, based on their clinical relevance to the population, previously reported associations with the dependent variables, and data availability (5, 7, 8, 11, 31). These variables included data on demographics, symptoms, physical function, self-reported physical activity before COVID-19, mental health, and self-rated health, and are presented together with the dependent variables in Table I. Missing data, assumed to be missing at random, were imputed prior to conducting the regression analyses using the “missForest” package in R, which produces low imputation errors for continuous and categorical variables (32). For further variable selection, given the large number of potential independent variables relative to the number of observations, relaxed lasso with cross-validation, using the “glmnet” package in R, were employed (33). The mean squared error (MSE) served as the performance criterion for the linear models, while the deviance was used for the logistic model. The optimal lambda value, corresponding to the minimum MSE or deviance, was selected. Subsequently, the multiple linear and logistic regression models were constructed, incorporating all independent variables selected by the relaxed lasso simultaneously. This approach promoted model tuning to identify independent associations, balancing accuracy and simplicity, thereby enhancing robustness and validity. Assumption checks were performed both numerically and visually. To assess the magnitude, and to facilitate interpretation and comparison of associations, we reported both unstandardized (b) and standardized coefficients (β) in the multiple linear regression models (small: 0.1; moderate: 0.3; large: 0.5). In the logistic regression analyses, effect sizes were expressed as odds ratios (OR) (small: 1.5; moderate: 2.5 large: 4.0).

**Table I:** Overview of the physical activity outcomes used as dependent variables, and the potentially associated variables.

**Dependent variables**
Steps/day
Brisk walking minutes/day
Awake sedentary minutes/day
Low-activity/prolonged-sedentary (<5,000 steps, <22 brisk walk min, >8 h sedentary)
**Potentially associated variables**
Demographics
Sex (female/male)
Age (in years at assessment)
Body Mass Index (BMI) in kg/m² at assessment
Higher education (>12 years)
Number of previous comorbidities (before COVID-19)
Work/Study > 50%, before COVID-19 and at assessment
Hospitalized due to COVID-19
Months since COVID-19
POTS diagnosis related to COVID-19
Self-reported symptoms
Number of self-reported symptoms at assessment
Self-reported symptoms with prevalence ≥ 20%^a^ (Chest pressure, Concentration difficulties, Cough, Difficulties taking deep breaths, Fatigue, Fever, Headache, Insomnia, Musculoskeletal pain, Palpitations, Paraesthesia, Post-exertional symptom exacerbation, Weight gain)
Physical function
6-minute walk test (% of predicted walk distance)
1-minute sit-to-stand test (% of predicted number of sit-to-stands in 1 minute)
Hand grip strength (% of predicted value)
Maximal inspiratory pressure (% of predicted value)
Forced expiratory volume in 1 s (% of predicted value)
Forced vital capacity (% of predicted value)
mMRC Score ≥ 2 (Indictive of clinically relevant dyspnoea)
Self-reported physical activity level
Frändin/Grimby activity score before COVID-19
Mental health
Patient Health Questionnaire 9 (PHQ-9) ≥10 (Indictive of moderate severe depression)
General Anxiety Disorder 7-item scale (GAD-7) ≥10 (Indictive of moderate severe anxiety)
Self-rated health
EQ VAS (0-100)

Potentially associated variables, to use as independent variables, were selected based on clinical relevance, data availability, and previously reported associations in individuals with post-COVID-19 condition. The potentially associated variables were then used in further selection of independent variables, through relaxed lasso, to construct the regression models.

a Self-reported symptom of dyspnoea excluded as mMRC dyspnoea scale was included.

## Results

### Participants

**Fig. 1:**
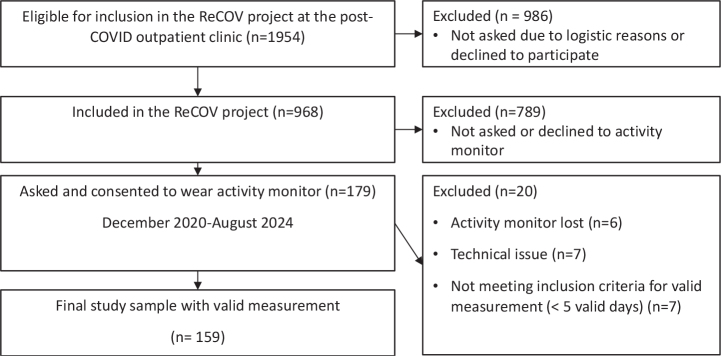
Participant flowchart illustrating the stages of participant selection.

**Fig. 2:**
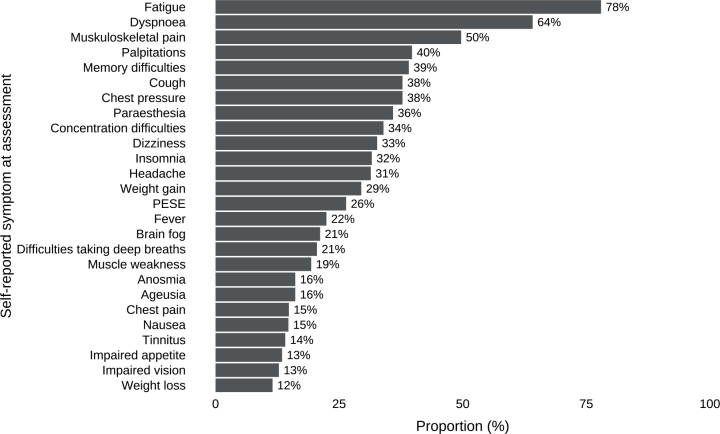
Most common self-reported symptoms. The most common self-reported symptoms (prevalence ≥ 10%) presented in a bar chart, reported by participants with post-COVID-19 condition at the time of physical activity and sedentary behaviour assessment using activity monitors (*n* = 159).

Of the 179 participants who consented to wear an activity monitor, 20 were excluded for various reasons as seen in a flowchart in Fig. 1. The remaining 159 participants wore the monitor for a median of 8 valid days (IQR 8–9). As detailed in Table II, the mean age of the participants was 50 years, 64% were women, 34% had been hospitalized due to COVID-19, and 19% had a diagnosis of POTS. Prior to COVID-19, most participants were employed or studying, reported high levels of physical activity, and had no functional limitations according to the post-COVID-19 functional status scale. The most frequently reported symptoms were fatigue (78%), dyspnoea (64%), and musculoskeletal pain (50%) (Fig. 2).

**Table II:** Characteristics of the study participants

Characteristics	Total sample	Sub-groups	
		Low-activity/prolonged-sedentary^a^	Others
Number of participants	159 (100%)	46 ( 29%)	113 (71%)
Sex, female	102 (64%)	29 (63%)	73 (65%)
Age (years)	50 (13)	51 (13)	50 (13)
BMI (kg/m^2^)	27 (23–30)	27 (23–31)	27 (23–30)
Higher education (> 12 years)	114 (86%)^26^	36 (88%)^5^	78 (85%)^21^
Employment rate (work/study > 50%)			
Before COVID-19	132 (83%)	39 (85%)	93 (82%)
At assessment	52 (33%)	20 (43%)	32 (28%)
Current smoker	5 (3%)	1 (2%)	4 (4%)
No. of previous comorbidities	2 (1–3)	2 (1–3)	1 (0–2)
Hospitalized due to COVID-19	57 (36%)	15 (33%)	42 (37%)
Months since COVID-19	15 (11–24)	16 (11–24)	15 (11–23)
POTS diagnosis after COVID-19	31 (19%)	16 (35%)	15 (13%)
No. of PCC symptoms	9 (5–13)	11 (7–15)	9 (5–13)
Post-COVID-19 functional status (PCFS)			
Before COVID-19	0 (0–0)^10^	0 (0–0)^4^	0 (0–0)^6^
At assessment	3 (2–3)^6^	3 (2–3)^3^	2 (1–3)^3^
Physical function			
6-min walk test (%)	90 (75–101)^3^	83 (66–96)^2^	91 (77–103)^1^
1-min sit-to-stand test (%)	65 (52–81)^9^	61 (46–70)^3^	66 (54–84)^6^
Hand grip strength	102 (84–115)^5^	101 (77–112)^2^	102 (87–120)^3^
Maximal inspiratory pressure (%)	94 (75–110%)^4^	93 (77–107%)^1^	94 (72–111%)^3^
FEV_1_ (%)	88 (79–96)^28^	89 (79–97)^11^	88 (79–95)^17^
FVC (%)	86 (78–93)^28^	84 (75–93)^11^	87 (78–93)^17^
mMRC dyspnoea	2 (1–2)^10^	2 (1–3)^1^	1 (1–2)^9^
mMRC dyspnoea ≥ 2	76 (51%)^10^	29 (63%)^1^	47 (42%)^9^
Self-reported physical activity (Frändin/Grimby)			
Before COVID-19	5 (4–5)^3^	4 (4–5)^1^	5 (4–5)^2^
At assessment	3 (2–4)^5^	2 (2–3)^1^	3 (2–4)^4^
Mental health			
PHQ-9 score	9 (5–14)^17^	11 (8–15)^3^	9 (5–13)^14^
PHQ-9 ≥ 10	67 (47%)^17^	24 (56%)^3^	43 (43%)^14^
GAD-7 score	5 (2–9)^21^	4 (2–10)^3^	5 (2–8)^18^
GAD-7 ≥ 10	32 (23%)^21^	13 (30%)^3^	19 (20%)^18^
Self-rated health			
EQ VAS	50 (35–70)^19^	40 (31–69)4	55 (40–70)^15^

Characteristics of participants with post-COVID-19-condition in the total study sample, in the sub-group with low activity combined with prolonged sedentary time and others who did not belong to this sub-group. Values are presented as mean (SD), median (IQR), or n (%). Missing values are reported using superscript after each value, if present.

a < 5,000 steps, < 22 brisk walking min, and > 8 sedentary h.

FEV^1^: forced expiratory volume in 1 sond; Frändin/Grimby: Frändin/Grimby activity scale; FVC: forced vital capacity; GAD-7: General Anxiety Disorder 7-item scale; mMRC dyspnoea: Modified Medical Research Council Dyspnea Scale; PHQ-9: Patient Health Questionnaire; POTS: postural orthostatic tachycardia syndrome.

**Fig. 3:**
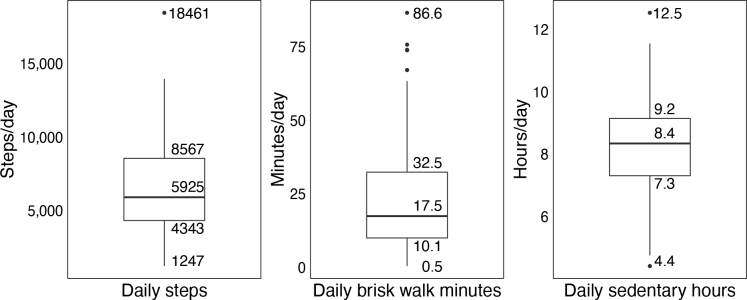
Description of daily physical activity and sedentary time for the total sample. Boxplots describing the daily number of steps, brisk walking time, and sedentary time for the total study sample of participant with post-COVID-19 condition (*n* = 159). Values are presented as medians, quartiles, minimum, and maximum.

**Fig. 4:**
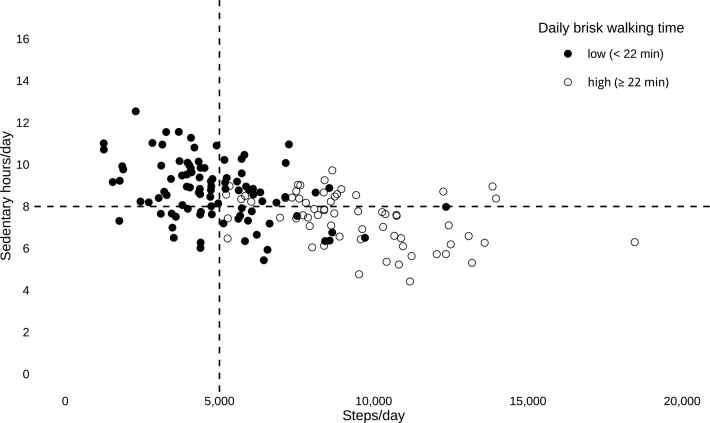
Illustration of daily physical activity and sedentary time for each participant. A descriptive plot illustrating the daily number of steps, brisk walking time, and sedentary time for each participant with post-COVID-19 condition in the total study sample. Dashed lines indicate the cut-off values for daily steps (5,000) and sedentary time (8 h). Filled circles represent participants with low brisk walking time (< 22 min), while open circles represent those with high brisk walking time (> 22 min). Dots in the upper left quadrant represent the 46 participants exhibiting low physical activity combined with prolonged sedentary time, defined as taking fewer than 5,000 steps, engaging in less than 22 min of brisk walking, and spending more than 8 h being sedentary per day.

### Steps, brisk walking time and sedentary time

In the total study sample, 36% (*n* = 58) took fewer than 5,000 steps per day, 60% (*n* = 96) engaged in less than 22 min of brisk walking, and 57% (*n* = 90) were sedentary for more than 8h during their waking hours. Some 29% (*n* = 46) of the participants exhibited both low physical activity and prolonged sedentary behaviour (Figs 3 and 4).

### Factors associated with physical activity and sedentary time

**Fig. 5:**
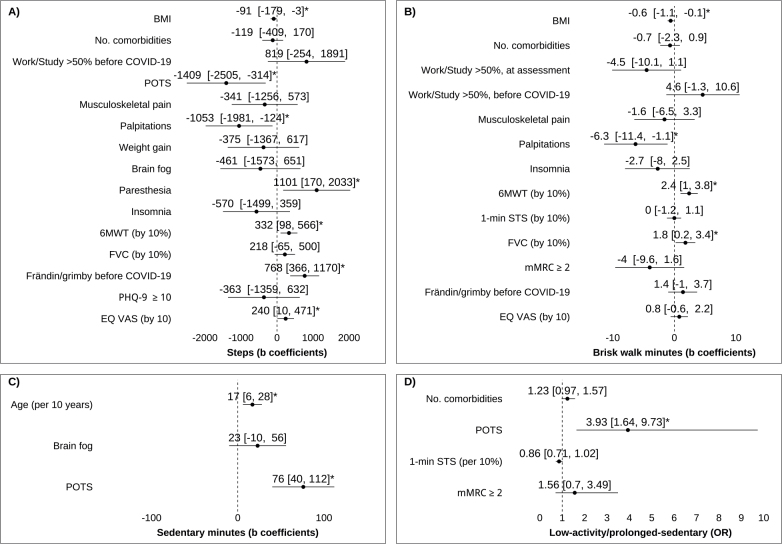
Forest plots of factors associated physical activity and sedentary time. Forest plots in 4 panels (A–D) presenting the results of multiple linear and logistic regression analyses identifying factors associated with (A) daily steps, (B) brisk walking time, (C) sedentary time, and (D) exhibiting low physical activity combined with prolonged sedentary time among participants with post-COVID-19 condition. Unstandardized regression coefficients (b) and odds ratios (OR) are shown with 95% confidence intervals (CI) and *p*-values. Statistically significant associations (*p* < 0.05) are marked with an asterisk (*). Model for steps: F(15, 143) = 5.821; *p* < 0.001; R² = 0.38; adjusted R² = 0.31.Model for brisk walking time: F(13, 145) = 6.058; p < 0.001; R² = 0.35; adjusted R² = 0.29. Model for sedentary time: F(3, 155) = 8.121; *p* < 0.001; R² = 0.14; adjusted R² = 0.12. Model for low activity/prolonged sedentary behaviour: log-likelihood test: χ² = 20.314, *p* < 0.001; Nagelkerke R² = 0.17; AUC = 0.70. Frändin/Grimby: Frändin/Grimby activity scale; mMRC dyspnoea: Modified Medical Research Council Dyspnea Scale; PHQ-9: Patient Health Questionnaire; POTS: postural orthostatic tachycardia syndrome.

Having POTS (β = −0.18), palpitations (β = −0.17), and a higher BMI (β = −0.16) were associated with taking fewer steps. Conversely, symptoms of paraesthesia (β = 0.17), a greater walking distance in the 6MWT (β = 0.22), higher pre-COVID-19 activity levels on the Frändin/Grimby scale (β = 0.26), and better self-rated health on the EQ VAS (β = 0.16) were associated with taking more steps. Having symptom of palpitations (β = −0.18) and higher BMI (β = −0.19) were associated with spending less time in brisk walking, whereas a greater walking distance in the 6MWT (β = 0.28) and better lung function (forced vital capacity; β = 0.16) were associated with spending more time in brisk walking. POTS (β = 0.33) and higher age (β = 0.24) were associated with more sedentary time. Having POTS was also the only independent variable associated with exhibiting both low physical activity and prolonged sedentary time (OR = 3.93) (Fig. 5).

## DISCUSSION

In this cross-sectional study, we found that a considerable proportion of participants with PCC showed low physical activity levels and prolonged sedentary time at 15 months after COVID-19. Notably, nearly one-third exhibited a combination of both behaviours. Additionally, we identified several associated factors of possible clinical relevance. POTS was associated with both reduced physical activity and increased sedentary time, while palpitations were associated with reduced physical activity. Conversely, a greater walking distance in the 6MWT and better lung function (forced vital capacity), previous activity levels, and self-rated health were associated with increased physical activity.

Perhaps the most clinically relevant result is that nearly a third (29%) of the participants exhibited low physical activity levels combined with prolonged sedentary time, a finding not previously reported in PCC. This suggests that a significant proportion of individuals with PCC may face a greater risk of further long-term health issues (9–11). These results are comparable to recent observations in individuals with stroke, where 33% were found to exhibit a similar pattern (34). There was large variability in the observed daily number of steps, brisk walking time, and sedentary time across the study sample, indicating a heterogeneous group with diverse activity profiles. This heterogeneity underscores the importance of individualized assessment and intervention strategies when addressing physical activity and sedentary behaviour in individuals with PCC. In contrast to our findings – which revealed relatively low levels of physical activity, with a median of 5,925 steps/day and 17.5 min of brisk walking – Rosa-Souza et al. (2024) reported a considerably greater number of daily steps (7,496 steps/day) and a longer duration of MVPA (30 mins/day) among previously hospitalized and non-hospitalized individuals with PCC at 15 months post-infection (7). The discrepancies in step count may reflect differences in the activity monitors used and study populations, with the former study possibly including individuals with less severe PCC and excluding those with more severe symptoms and functional limitations (7). Conversely, the observed brisk walking time is comparable to the duration of MVPA reported by Plekhanova et al. (5) – 14.9 min/day for women and 21.1 min/day for men – 8 months post-discharge in previously hospitalized individuals, and by Silva et al. (6), who reported 13.6 min/day 2 months following mild to moderate COVID-19. However, making a precise comparison between brisk walking and MVPA, as used in previous studies, is not possible, as MVPA may encompass a broader range of activities, whereas brisk walking – although a common form of physical activity at moderate intensity for most individuals – represents only 1 specific type. Compared with other populations, the daily steps and brisk walking time found in this study are similar to those reported in chronic obstructive pulmonary disease, stroke, and severe chronic heart failure (34–36). In contrast, we found considerably fewer steps and lower brisk walking time compared with healthy adults in the Nordic countries, who have been shown to accumulate 7,451–8,338 steps/day and 33–46 min/day of MVPA (37). The observed high proportion of participants who engaged in less than 22 min of brisk walking per day (60%), equating to under 150 min per week, likely does not meet the recommended levels of physical activity (11). This is comparable to the 70% not meeting the recommended 150 min of MVPA per week reported by Rosa-Souza et al. (2024) in a similar PCC population, but substantially higher than the 8–10% of healthy Swedish adults who self-reported not meeting the recommended levels (7, 38). However, caution is advisable, as this involves a comparison with MVPA time and self-reported data. The daily sedentary time observed in this study is similar to that reported in previous studies involving individuals with PCC, as well as in comparisons with healthy adults in the Nordic countries (6, 7, 37). The observed high proportion of individuals (57%) with prolonged sedentary time is expected, given the nature of the condition. However, it remains concerning from a long-term health perspective – particularly in populations such as this one, characterized by low levels of physical activity (11).

We also identified several factors associated with physical activity and sedentary behaviour in PCC, which are important to consider. The associations between POTS and taking fewer daily steps, and increased sedentary time, as well as exhibiting low physical activity combined with prolonged sedentary time have not previously been described. The observed associations showed small to moderate effect sizes, with some uncertainty in precision, especially for daily steps and the combined outcome. These findings suggest that having POTS may negatively impact both physical activity and sedentary time, probably due to common symptoms such as orthostatic intolerance, fatigue, post-exertional symptom exacerbation, and exercise intolerance (4). The observed associations between higher levels of physical function, as measured by the predicted distance in the 6MWT, and a greater number of steps and increased brisk walking time are not surprising. These associations were characterized by small to moderate effect sizes, with relatively narrow confidence intervals, suggesting good precision. While the cross-sectional design limits causal inference, the consistency and strength of these associations support their potential clinical relevance. The previous high self-reported physical activity level before COVID-19, associated with the current number of steps taken, suggests lasting benefits of a previously active lifestyle. This association was characterized by a moderate effect size and good precision. Regarding self-reported symptoms, the associations between palpitations and fewer steps and decreased brisk walking time have not previously been reported in PCC. These associations were characterized by small effect sizes and relatively narrow confidence intervals. Possible explanations could include discomfort during activity and underlying health conditions that may impair the ability to engage in physical activity. Furthermore, paraesthesia was found to be associated with an increased number of steps. Although this association showed a small effect size and good precision, the finding is unexpected and should be interpreted with caution. It could possibly be attributed to an increase in symptoms in more active individuals, potentially due to a dysfunction in physiological adaptation to physical activity (39). Surprisingly, fatigue and post-exertional symptom exacerbation were not identified as associated factors in our analysis, contrary to previous findings (7, 8). These discrepancies could be explained by differences in assessments and statistical modelling. Different types of activity monitors and questionnaires were used to assess fatigue and post-exertional symptom exacerbation in the previous studies, whereas our study relied on self-reported symptoms. Additionally, the high prevalence of fatigue in our sample may have limited the variability needed to detect a statistical association. Demographic factors also played a role, with increased BMI being associated with lower levels of physical activity and sedentary time increasing with age. Population-based studies from the Nordic countries have shown similar trends, indicating that older adults tend to spend more time being sedentary (37).

Improving physical activity in PCC is crucial for long-term public health, and exercise, as part of physical activity, is important for rehabilitation in PCC (11, 31). Increasing time in MVPA among those exhibiting low activity levels combined with prolonged sedentary time could potentially attenuate the long-term health risks (9). However, managing these behaviours in this population is complex and requires consideration of various factors such as POTS and post-exertional symptom exacerbation (7, 40). Although the optimal strategy remains undetermined, this study highlights the need for comprehensive assessments to ensure safe and effective recommendations (31). Clinical expertise suggests that exercise, as part of interprofessional rehabilitation, must be tailored to individual needs. When physical activity or exercise induces post-exertional symptom exacerbation, careful monitoring, pacing, and symptom titration are essential (31, 40). Furthermore, it is crucial to address other aspects of life, involving interdisciplinary rehabilitation teams for individuals with severe PCC (31, 40). Research going forward should target high-risk groups with complex symptomatology, include longitudinal follow-up to monitor changes, and evaluate interventions that improve physical activity without exacerbating symptoms in the long term.

Due to the cross-sectional design, we cannot infer causality between the associated factors and the outcome variables. Our study sample includes only individuals with PCC attending a specialized post-COVID clinic during specific periods, which may affect representativeness and generalizability. Although the sampling method was feasible in the clinical setting, it could introduce bias. However, the selection of participants was random during data collection periods. While our identified associations provide valuable insights of possible clinical relevance, some estimates have wide confidence intervals, thereby increasing uncertainty. Beyond data on previous comorbidities, POTS, and common symptoms (e.g., musculoskeletal pain), we did not collect information on other newly developed diseases or injuries that could potentially affect physical activity, which can be considered a limitation of the study. The activPAL, although not validated in PCC, has proved valid in other populations for measuring sedentary time and steps (37, 38). As the activPAL more accurately measures step cadence rather than intensity, we chose to report brisk walking instead of MVPA (38). However, this makes it challenging to compare our results with studies that have used other activity monitors. We defined brisk walking time using an absolute cadence threshold, which may not reflect moderate intensity for all individuals. To account for relative intensity, we recognize the need to validate cadence cut points against physical capacity. Physical activity was measured only through walking, as it represents the primary form of activity for most people. However, other types of exercise participants may have engaged in, such as cycling or swimming, were not captured, which constitutes another limitation of the study. Identifying awake periods based on activity patterns is inherently challenging, especially for individuals with irregular sleep schedules. Although sleep diaries were not used, the criteria applied seemed to identify waking hours with reasonable accuracy, even during nighttime. Additionally, retrospective self-reports of pre-COVID physical activity, assessed using the Frändin/Grimby activity scale, may be affected by recall bias. Data collection in a clinical setting during the pandemic led to some missing values. However, the overall extent of missing data was small, and the study included comprehensive assessments overall.

In conclusion, a significant proportion of individuals with PCC exhibited low physical activity levels combined with prolonged sedentary time, which could lead to further negative health consequences. The identified associations highlight the importance of considering factors such as comorbidity in POTS, physical impairments, and common symptoms such as palpitations when managing these behaviours. This study underscores the need for comprehensive assessments to ensure safe and effective strategies for improving physical activity, while addressing the complex needs of individuals with PCC.
